# The effect of seed location on functional connectivity: evidence from an image-based meta-analysis

**DOI:** 10.3389/fnins.2023.1120741

**Published:** 2023-05-31

**Authors:** Meng-Ting Li, Jia-Wei Sun, Lin-Lin Zhan, Collins Opoku Antwi, Ya-Ting Lv, Xi-Ze Jia, Jun Ren

**Affiliations:** ^1^School of Psychology, Zhejiang Normal University, Jinhua, China; ^2^Department of Clinical Neuroscience, Division of Neuro, Karolinska Institutet, Stockholm, Sweden; ^3^School of Western Studies, Heilongjiang University, Harbin, China; ^4^Center for Cognition and Brain Disorders, The Affiliated Hospital, Hangzhou Normal University, Hangzhou, China

**Keywords:** seed-based functional connectivity, seed selection, image-based meta-analysis, reproducibility, default mode network

## Abstract

**Introduction:**

Default mode network (DMN) is the most involved network in the study of brain development and brain diseases. Resting-state functional connectivity (rsFC) is the most used method to study DMN, but different studies are inconsistent in the selection of seed. To evaluate the effect of different seed selection on rsFC, we conducted an image-based meta-analysis (IBMA).

**Methods:**

We identified 59 coordinates of seed regions of interest (ROIs) within the default mode network (DMN) from 11 studies (retrieved from Web of Science and Pubmed) to calculate the functional connectivity; then, the uncorrected *t* maps were obtained from the statistical analyses. The IBMA was performed with the *t* maps.

**Results:**

We demonstrate that the overlap of meta-analytic maps across different seeds’ ROIs within DMN is relatively low, which cautions us to be cautious with seeds’ selection.

**Discussion:**

Future studies using the seed-based functional connectivity method should take the reproducibility of different seeds into account. The choice of seed may significantly affect the connectivity results.

## 1. Introduction

Our brain is a network of spatially distributed but functionally linked regions. Increasingly, and rightly, studies have treated the brain as an integrative network with regions neatly interacting functionally, using resting-state functional connectivity (Van den Heuvel and Hulshoff Pol, 2010). Resting-state functional connectivity (rsFC) refers to temporal dependence of neuronal activity patterns of anatomically separated brain regions when resting (Aertsen et al., 1989; Friston et al., 1993; Biswal et al., 1995). Relevant studies contribute to our understanding of this spontaneous activity, which forms communication across brain regions (Rosazza et al., 2012).

Seed-based functional connectivity was the first method employed to identify the resting state networks and provided a direct way to examine the regions with robust functional connectivity with the seed ROI (Cole et al., 2010; Smitha et al., 2017). The mean resting-state time series from the seed region correlated with the time series from other voxels of the entire brain results in a functional connectivity map (FC map). Thus, one could obtain the rsFC information of any meaningful voxel or brain region from the FC map. The map provides an elegant way to examine functional connectivity in the human brain (Van den Heuvel and Hulshoff Pol, 2010). This fundamental method has been applied in many studies (e.g., Cordes et al., 2000; Fox et al., 2005; Ji et al., 2020). In particular, it has been widely deployed in disease prognostication. The findings reveal considerable variability in neuropsychiatric illnesses, including Parkinson’s disease (Bi et al., 2020, 2021), schizophrenia (Yang et al., 2018), depression (Yu et al., 2020) and migraine (Wei et al., 2020). Notably, to perform seed-based FC, the seed (i.e., ROI) needs to be defined. Specifically, the seed ROI can be determined by activating the relevant task or using prior anatomical knowledge or standard brain atlases (Wu et al., 2018). However, there is no gold standard for selecting the seed. Given the multiplicity of available options (Sohn et al., 2015), the reproducibility across different seed ROIs is uncertain.

Cordes et al. (2000) noted that not every selected seed voxel yields the same functional connectivity map. However, whether and to what extent different seeds will significantly influence the results of the seed-based functional connectivity is unclear. The default mode network, the most fundamental resting-state network, is observed to be active even when people are not engaged in any goal-directed cognitive activities and show a deactivation pattern (Shulman et al., 1997; Bonnelle et al., 2012). The DMN is consist of medial prefrontal cortex (MPFC), posterior cingulate cortex/precuneus (PCC/PrC) and bilateral inferior parietal lobules (IPL) (Assaf et al., 2010). Zuo and Xing (2014) conducted a multisite analysis of resting-state metrics’ test–retest reliability. They found that the default mode network was one of the most reliable ones within the seven brain networks, including visual, somatomotor, dorsal attention, limbic, frontoparietal and DMN networks (Yeo et al., 2011). Sex differences were chosen to identify the statistical difference in the functional connectivity because sex can be easily tested across large scale datasets in the light of its objective nature (Chen et al., 2018). Meanwhile, the functional differences in the human brain between men and women have been well investigated in previous resting-state studies. Allen et al. (2011) explored the effect of sex on the resting state networks (RSNs) and found females generate stronger intra-network connectivity and meles show stronger inter-network connectivity. Beltz et al. (2015) investigate the sex-related differences in resting state brain function of smokers and identified greater connectivity within the DMN in women and greater connectivity within the reward network in men. Evidences from these studies supported the importance of sex differences in resting state studies.

The present study aims two-fold: first, to identify whether the results contained in functional connectivity studies using different seeds are reproducible; second, to quantify the effect of the spatial location of seed ROIs on the seed-based functional connectivity.

## 2. Materials and methods

### 2.1. Participants and imaging protocols

MRI data were obtained from the Consortium for Reliability and Reproducibility (CoRR) (Zuo and Xing, 2014). The 36 datasets from the CoRR originally included 1725 participants who underwent at least two scanning sessions. Here, we only chose the baseline RS-fMRI data to analyze the functional connectivity difference between males and females. With the above exclusion criteria, we made quality control. From this perspective, 1,290 participants from 30 datasets were selected (age 25.797 ± 15.538, 671 females, see Table 1 for details). Six datasets were excluded due to the loss of information or the small sample size of participants. Participants from the rest of the datasets were excluded if their head motion was excessive (more than 2.5 mm of maximal translation in any direction of x, y, or z or 2.5°of maximal rotation throughout scanning). To control the confounds of handedness, participants with non-right handedness were excluded. Participants with low-quality normalization or incomplete brain coverage were excluded. In addition, age was matched (*p* > 0.5, two sample *T*-test) between male and female groups in each dataset.

**Table 1:** Sample characteristics of CoRR dataset.

Dataset	Sample size (N, male/female)	Mean age (Years, M ± SD)	Age *p*
BMB_1	48 (23/25)	30.721 ± 7.200	0.792
BNU_1	53 (29/24)	23.321 ± 2.137	0.549
BNU_2	59 (33/26)	21.259 ± 0.795	0.502
BNU_3	43 (21/22)	22.302 ± 1.489	0.593
HNU_1	28 (15/13)	24.250 ± 2.205	0.770
IACAS	24 (24/11)	26.167 ± 3.908	0.906
IBA_TRT	32 (16/16)	25.969 ± 6.660	0.697
IPCAS_1	23 (5/18)	20.522 ± 1.675	0.685
IPCAS_2	31 (11/20)	13.355 ± 0.877	0.647
IPCAS_3	25 (4/21)	20.560 ± 1.557	0.549
IPCAS_4	18 (9/9)	23.278 ± 1.602	0.888
IPCAS_7	57 (19/38)	12.421 ± 2.719	0.610
IPCAS_8	7 (2/5)	57.143 ± 3.288	0.540
JHNU_1	25 (20/5)	23.640 ± 3.807	0.592
LMU_1	24 (12/12)	24.542 ± 1.719	0.730
LMU_2	40 (18/22)	50.825 ± 22.456	0.855
LMU_3	25 (16/9)	69.800 ± 7.927	0.791
MPG_1	15 (8/7)	24.800 ± 1.521	0.858
MRN_1	38 (20/18)	24.947 ± 11.409	0.954
NKI_2	36 (9/27)	13.167 ± 2.635	0.616
NYU_1	10 (3/7)	25.900 ± 4.067	0.562
NYU_2	164 (105/59)	19.979 ± 10.809	0.577
SWU_1	19 (6/13)	21.632 ± 1.770	0.746
SWU_2	22 (7/15)	20.772 ± 1.601	0.660
SWU_3	22 (7/15)	20.409 ± 1.681	0.971
SWU_4	221 (110/111)	19.982 ± 1.179	0.649
UM	71 (20/51)	65.930 ± 6.332	0.792
UPSM_1	71 (35/36)	15.528 ± 2.856	0.800
UWM	19 (12/7)	24.684 ± 3.110	0.746
XHCUMS	20 (13/7)	50.700 ± 6.018	0.702
Gender *p*	0.727		

### 2.2. Data preprocessing

All preprocessing of resting-state fMRI data was processed using *RESTplus* V1.24 (Jia et al., 2019). Initially, the first 10 time points were discarded to overcome the influence of instability when the scanner was switched on while participants adapted to the scanner’s noise. Second, a slice-timing correction was performed to correct the acquisition time delay for all volumes. Third, head motion correction. Fourth, individual structural images were co-registered to mean functional images; then, the co-registered structural images were segmented into gray matter (GM), white matter (WM), cerebrospinal fluid (CSF), bone, soft tissue and air/background (Kazemi and Noorizadeh, 2014). Parameters generated from step 4 were used to apply for functional images spatially normalized to Montreal Neurologic Institute space (the resampling voxel size = 3 mm × 3 mm × 3 mm). Fifth, normalized fMRI data were smoothed with a Gaussian kernel of 6 mm × 6 mm × 6 mm full-width at half maximum (FWHM). Sixth, the linear trend of the time course was removed. Next, head motion effects (using Friston 24 parameters) (Friston et al., 1996), global mean signals and white matter signals were regressed out to minimize confounds. Finally, data were temporally band-pass filtered (0.01 Hz – 0.08 Hz).

### 2.3. Functional connectivity calculation

Fifty-nine coordinates within DMN were extracted from the 11 studies, and all coordinates were converted to Montreal Neurological Institute space. As for resting-state functional connectivity, we defined fifty-nine spherical regions of interests (ROIs) with a radius of 6 mm centered on the fifty-nine coordinates. The radius was defined in line with a prior study (Yu et al., 2020). After data preprocessing, the time course of each seed ROI was extracted, and functional connectivity was calculated by computing the Pearson correlation coefficient between the mean signal time course from the ROI and all other voxels in the entire brain.

## 3. Statistical analysis

To increase the normality of the distribution of correlation, all FC maps were transformed from *r* values to *z* values through Fisher’s *r*-to-*z* transformation. Further, two-sample *t*-tests were performed using Data Processing & Analysis for Brain Imaging (DPABI) (Yan et al., 2016) to compare the difference between males and females’ FC maps, which Fisher’s *r*-to-*z* transformation has processed. Finally, all *t* maps were used for subsequent image-based meta-analysis.

Meanwhile, to show the functional connectivity pattern of each seed in different gender, for each seed, we also calculated the one sample *t* test results of FC maps of different genders with center as covariate (Results maps see Supplementary Figures S2–S60 in the supplementary material).

### 3.1. Literature search

A literature search of relevant articles was conducted in Web of Science and PubMed as of December 13, 2020, using the keywords “default mode network” or “DMN.” First, the titles of highly cited papers on the topic labeled by the databases were recorded. Then, a full-text search for these articles was performed to assess the documents for those that provided coordinates of DMN nodes. Finally, the reference lists of articles with coordinates of DMN nodes were manually scanned for other articles that were not retrieved at the database search phase. When multiple articles reported exact coordinates, the earliest one was included. And if the coordinates reported in the papers were quoted, the source article was selected instead. When the coordinates of the same article were inconsistent in different references, the coordinates reported in the original research were included. As a result, 68 articles were identified by our search. These criteria generated 59 coordinates of DMN nodes from 11 articles that were retrieved from our searches.

### 3.2. Data extraction and coding

All coordinates were converted to Montreal Neurological Institute space using the tal2icbm transform (Lancaster et al., 2007). According to the converted coordinates, each seed was categorized into a brain region of anatomical automatic labeling (AAL) atlas (Tzourio-Mazoyer et al., 2002) (see Table 2 for details). Visualization of seeds and brain network were shown through the BrainNet Viewer software (Xia et al., 2013).

**Table 2:** Montreal Neurological Institute (MNI) coordinates of 59 DMN regions of interest (ROIs).

Study	ROI name	Region	Coordinates (x, y, z)
Lu et al. (2012)	Angular_L (aal)	Infer parietal ctx	(−49, −65, 35)
Shulman et al. (1997)	Angular_L (aal)	Lateral parietal	(−47, −66, 43)
Di et al. (2014)	Angular_L (aal)	Inferior parietal lobule	(−50, −63, 32)
Smigielski et al. (2019)	Angular_L (aal)	Angular gyri	(−50, −56, 30)
Lu et al. (2012)	Angular_R (aal)	Infer parietal ctx	(56, −61,24)
Baliki et al. (2014)	Angular_R (aal)	Lateral parietal	(46, −60, 32)
Shulman et al. (1997)	Angular_R (aal)	Inferior cortex	(50, −55, 38)
Di et al. (2014)	Angular_R (aal)	Inferior parietal lobule	(48, −69, 35)
Smigielski et al. (2019)	Angular_R (aal)	Angular gyri	(52, −52, 32)
Andrews-Hanna et al. (2010)	Calcarine_L (aal)	Rsp	(−14, −52, 8)
Baliki et al. (2014)	Cingulum_Ant_L (aal)	ACC	(2, 36, 22)
Andrews-Hanna et al. (2010)	Cingulum_Ant_L (aal)	aMPFC	(−6, 52, −2)
Sridharan et al. (2008)	Cingulum_Mid_L (aal)	PCC	(−7, −43, 33)
Di et al. (2014)	Cingulum_Post_L (aal)	PCC	(0, −52, 26)
Smigielski et al. (2019)	Cingulum_Post_L (aal)	PCC	(1, −36, 30)
Sharp et al. (2011)	Cingulum_Post_L (aal)	PCC	(−2, −46, 20)
Kucyi et al. (2014)	Cingulum_Post_L (aal)	PCC	(−8, −50, 28)
Lu et al. (2012)	Frontal_Inf_Orb_L (aal)	Orbiatal frontal ctx	(−49, 40, −11)
Shulman et al. (1997)	Frontal_Inf_Orb_L (aal)	Left infrior frontal cortex	(−35, 49, −16)
Lu et al. (2012)	Frontal_Inf_Orb_R (aal)	Orbiatal frontal ctx	(51, 34, −10)
Kucyi et al. (2013)	Frontal_Med_Orb_L (aal)	mPFC	(−2, 58, −6)
Sridharan et al. (2008)	Frontal_Med_Orb_L (aal)	vmPFC (11)	(−2, 36, −10)
Shulman et al. (1997)	Frontal_Med_Orb_L (aal)	Medial prefrontal cortex (10)	(0, 51, −14)
Smigielski et al. (2019)	Frontal_Med_Orb_L (aal)	MPFC	(−6, 44, −6)
Di et al. (2014)	Frontal_Med_Orb_R (aal)	MPFC	(3, 54, −2)
Shulman et al. (1997)	Frontal_Mid_L (aal)	Left dorsolateral frontal cortex	(−28, 34, 38)
Shulman et al. (1997)	Frontal_Sup_L (aal)	Dorsal-ventral axis	(−10, 49, 38)
Shulman et al. (1997)	Frontal_Sup_L (aal)	Dorsal-ventral axis	(−15, 63, 19)
Lu et al. (2012)	Frontal_Sup_Medial_L (aal)	MPFC/ACC	(2, 60, 26)
Baliki et al. (2014)	Frontal_Sup_Medial_L (aal)	mPFC	(−4, 58, 2)
Andrews-Hanna et al. (2010)	Frontal_Sup_Medial_L (aal)	dMPFC	(0, 52, 26)
Shulman et al. (1997)	Frontal_Sup_Medial_R (aal)	Dorsal-ventral axis-	(7, 57, 30)
Sharp et al. (2011)	Frontal_Sup_Medial_R (aal)	vMPFC	(2, 54, 8)
Shulman et al. (1997)	Frontal_Sup_Orb_L (aal)	Right prefrontal cortex	(−20, 63, −2)
Andrews-Hanna et al. (2010)	Fusiform_L (aal)	PHC	(−28, −40, −12)
Lu et al. (2012)	Hippocampus_L (aal)	Parahipp gyrus/hipp	(−26, −12, −23)
Baliki et al. (2014)	Insula_L (aal)	Inferior frontal gyrus	(−38, 10, −12)
Kucyi et al. (2013)	Occipital_Mid_L (aal)	Temporooccicipital junction	(−46, −76, 24)
Andrews-Hanna et al. (2010)	Occipital_Mid_L (aal)	pIPL	(−44, −74, 32)
Andrews-Hanna et al. (2010)	ParaHippocampal_L (aal)	HF+	(−22, −20, −26)
Lu et al. (2012)	ParaHippocampal_R (aal)	Parahipp gyrus/hipp	(28, −11, −25)
Shulman et al. (1997)	ParaHippocampal_R (aal)	Parahipp gyrus/hipp	(24, −10, −24)
Shulman et al. (1997)	Parietal_Inf_L (aal)	Inferior parietal cortex	(−55, −36, 47)
Lu et al. (2012)	Precuneus_L (aal)	PCC	(0, −57, 35)
Fransson (2005)	Precuneus_L (aal)	PCC	(0, −56, 30)
Shulman et al. (1997)	Precuneus_L (aal)	PCC	(−4, −47, 45)
Andrews-Hanna et al. (2010)	Precuneus_L (aal)	PCC	(−8, −56, 26)
Baliki et al. (2014)	Precuneus_R (aal)	PCC	(2, −56, 26)
Andrews-Hanna et al. (2010)	Rectus_L (aal)	vMPFC	(0, 26, −18)
Shulman et al. (1997)	Rectus_R (aal)	Inferior anterior cingulate	(4, 33, −19)
Baliki et al. (2014)	SupraMarginal_L (aal)	Supramarginal gyrus	(−56, −36, 26)
Andrews-Hanna et al. (2010)	SupraMarginal_L (aal)	TPJ	(−54, −54, 28)
Shulman et al. (1997)	Temporal_Inf_L (aal)	Left inferior temporal gyrus	(−52, −21, −22)
Andrews-Hanna et al. (2010)	Temporal_Inf_L (aal)	LTC	(−60, −24, −18)
Lu et al. (2012)	Temporal_Mid_L (aal)	Infer temporal gyrus	(−59, −6, −23)
Andrews-Hanna et al. (2010)	Temporal_Mid_L (aal)	TempP	(−50, 14, −40)
Lu et al. (2012)	Temporal_Mid_R (aal)	Infer temporal gyrus	(61, −12, −22)
Kucyi et al. (2013)	Temporal_Mid_R (aal)	Temporooccicipital junction	(44, −64, 16)
Kucyi et al. (2013)	Lingual_R (aal)	PCC	(6, −56, 2)

aal, Anatomical Automatic Labeling; R/L, right or left; ACC, anterior cingulate cortex; aMPFC, anterior medial prefrontal cortex; PCC, posterior cingulate cortex.

### 3.3. SDM Meta-analysis

We performed an image-based meta-analysis named Anisotropic Effect-Size Signed Difference Mapping (AES-SDM, version 5.15) to examine the FC differences between male and female. The AES-SDM approach uses full statistical images as input and allows both positive and negative values of the same map to be preserved (Joaquim Radua et al., 2014). This approach has been found valid and well described in previous studies (see Peters et al., 2012; Radua et al., 2012; Joaquim Radua et al., 2014; Welton et al., 2015).

**Table 3:** The mean and median Dice coefficient of each seed ROI.

ROI number	ROI name (x, y, z, MNI)	Dice coefficient		ROI number	ROI name (x, y, z, MNI)	Dice coefficient	
		Mean	Median			Mean	Median
1	Angular_L (−49, −65,35)	0.355	0.167	31	Frontal_Sup_Medial_L (0,52,26)	0.320	0.112
2	Angular_L (−47, −66,43)	0.336	0.161	32	Frontal_Sup_Medial_R (7,57,30)	0.345	0.111
3	Angular_L (−50, −63,32)	0.339	0.155	33	Frontal_Sup_Medial_R (2,54,8)	0.336	0.105
4	Angular_L (−50, −56,30)	0.262	0.154	34	Frontal_Sup_Orb_L (−20,63, −2)	0.202	0.105
5	Angular_R (56, −61,24)	0.212	0.152	35	Fusiform_L (−28, −40, −12)	0.260	0.102
6	Angular_R (46, −60,32)	0.354	0.150	36	Hippocampus_L (−26, −12, −23)	0.259	0.100
7	Angular_R (50, −55,38)	0.291	0.148	37	Insula_L (−38,10, −12)	0.080	0.098
8	Angular_R (48, −69,35)	0.274	0.148	38	Occipital_Mid_L (−46, −76,24)	0.242	0.095
9	Angular_R (52, −52,32)	0.290	0.146	39	Occipital_Mid_L (−44, −74,32)	0.307	0.093
10	Calcarine_L (−14, −52, 8)	0.227	0.146	40	ParaHippocampal_L (−22, −20, −26)	0.156	0.085
11	Cingulum_Ant_L (2,36,22)	0.159	0.140	41	ParaHippocampal_R (28, −11, −25)	0.265	0.084
12	Cingulum_Ant_L (−6,52, −2)	0.320	0.140	42	ParaHippocampal_R (24, −10, −24)	0.275	0.084
13	Cingulum_Mid_L (−7, −43,33)	0.341	0.139	43	Parietal_Inf_L (−55, −36,47)	0.083	0.083
14	Cingulum_Post_L (0, −52,26)	0.337	0.138	44	Precuneus_L (0, −57,35)	0.326	0.081
15	Cingulum_Post_L (1, −36,30)	0.281	0.137	45	Precuneus_L (0, −56,30)	0.342	0.081
16	Cingulum_Post_L (−2, 46,20)	0.284	0.136	46	Precuneus_L (−4, −47,45)	0.272	0.078
17	Cingulum_Post_L (−8, −50,28)	0.330	0.136	47	Precuneus_L (−8, −56,26)	0.332	0.071
18	Frontal_Inf_Orb_L (−49,40, −11)	0.239	0.135	48	Precuneus_R (2, −56,26)	0.339	0.070
19	Frontal_Inf_Orb_L (−35,49, −16)	0.170	0.133	49	Rectus_L (0,26, −18)	0.249	0.066
20	Frontal_Inf_Orb_R (51,34, −10)	0.261	0.133	50	Rectus_R (4,33, −19)	0.276	0.054
21	Frontal_Med_Orb_L (−2,58, −6)	0.301	0.131	51	SupraMarginal_L (−56, −36,26)	0.089	0.053
22	Frontal_Med_Orb_L (−2,36, −10)	0.301	0.130	52	SupraMarginal_L (−54, −54,28)	0.176	0.047
23	Frontal_Med_Orb_L (0,51, −14)	0.309	0.130	53	Temporal_Inf_L (−52, −21, −22)	0.306	0.047
24	Frontal_Med_Orb_L (−6,44, −6)	0.329	0.129	54	Temporal_Inf_L (−60, −24, −18)	0.324	0.044
25	Frontal_Med_Orb_R (3,54, −2)	0.310	0.128	55	Temporal_Mid_L (−59, −6, −23)	0.281	0.044
26	Frontal_Mid_L (−28,34,38)	0.213	0.122	56	Temporal_Mid_L (−50,14, −40)	0.276	0.038
27	Frontal_Sup_L (−10,49,38)	0.300	0.121	57	Temporal_Mid_R (61, −12, −22)	0.341	0.037
28	Frontal_Sup_R (−15,63,19)	0.195	0.121	58	Temporal_Mid_R (44, −64,16)	0.114	0.032
29	Frontal_Sup_Medial_L (2,60,26)	0.345	0.116	59	Lingual_R (6, −56, 2)	0.122	0.030
30	Frontal_Sup_Medial_L (−4,58,2)	0.320	0.114				

For each center, AES-SDM constructs an effect size and corresponding variance map from the unthresholded two sample *T*-map. The method uses a standard random-effects model, which considers sample size, study precision and between-study heterogeneity, to combine the maps of different effect sizes and variance of different centers. The random-effects model ensures that studies with larger sample size or lower variability contribute more to the meta-analysis results. A randomization test that randomizes the location of the voxels within the SDM gray matter template was performed to assess the statistical significance. Since the corrected *p* value in fMRI articles is seriously affected by methodological factors, AES-SDM uses a combination of two thresholds, in which four uncorrected *p* values (*p* = 0.005, *p* = 0.001, *p* = 0.0005; *p* = 0.0001) are applied separately as the main threshold. In addition, a *Z*-based threshold is added to reduce the possibility of false-positive results (we set *z* > 1.00, which is the default setting of AES-SDM). Meanwhile, 10 voxels were applied to threshold the cluster size.

## 4. Results

### 4.1. Meta analysis results of gender difference

The meta-analytic maps of 30 centers in each seed were shared online.^1^

### 4.2. Consistency of meta-analytic results

**Figure 1:**
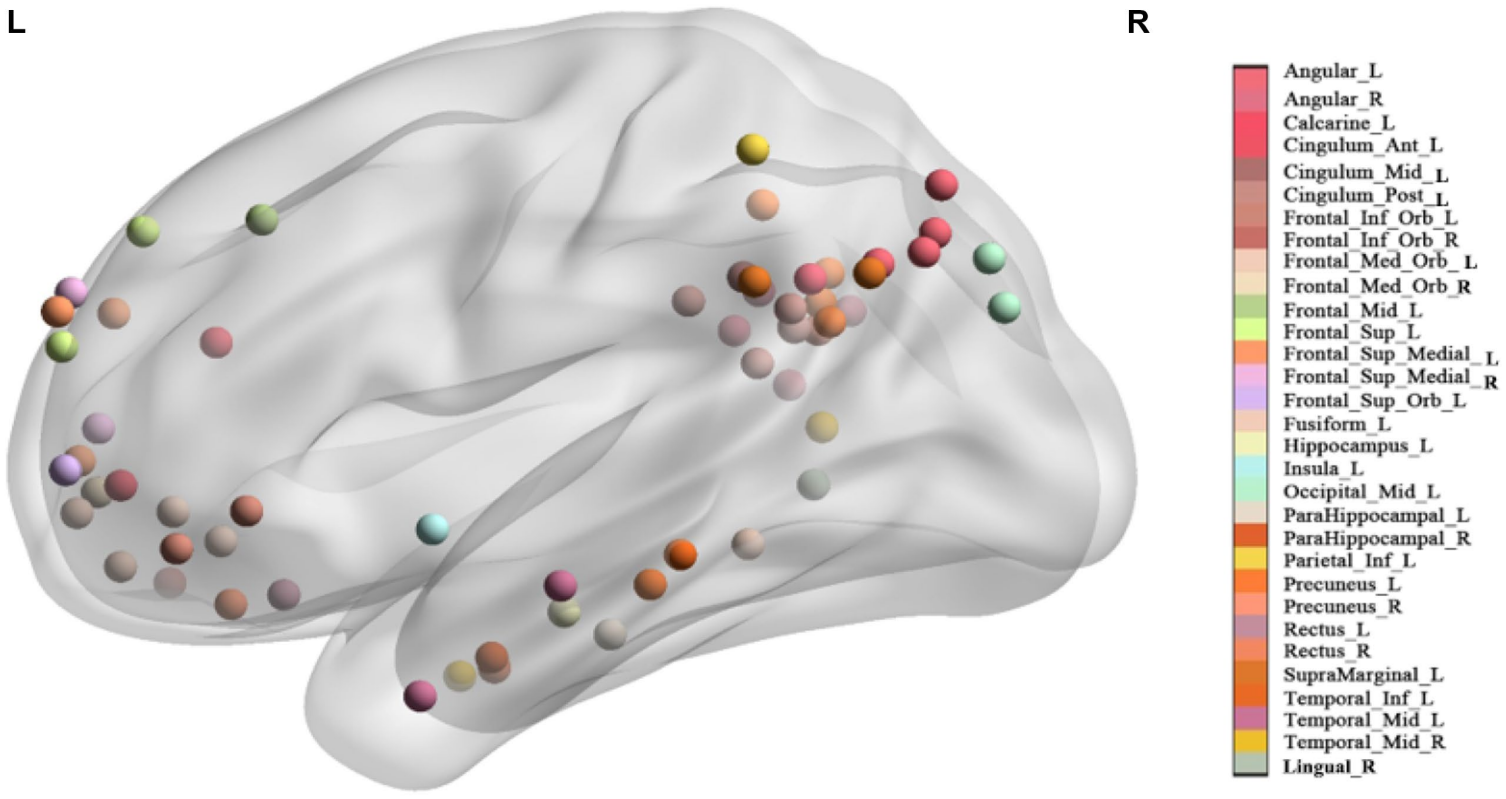
The location of each seed. Seeds with the same name are marked with the same color (See Supplementary Figure S1 in the supplementary material for multiple view).

**Figure 2:**
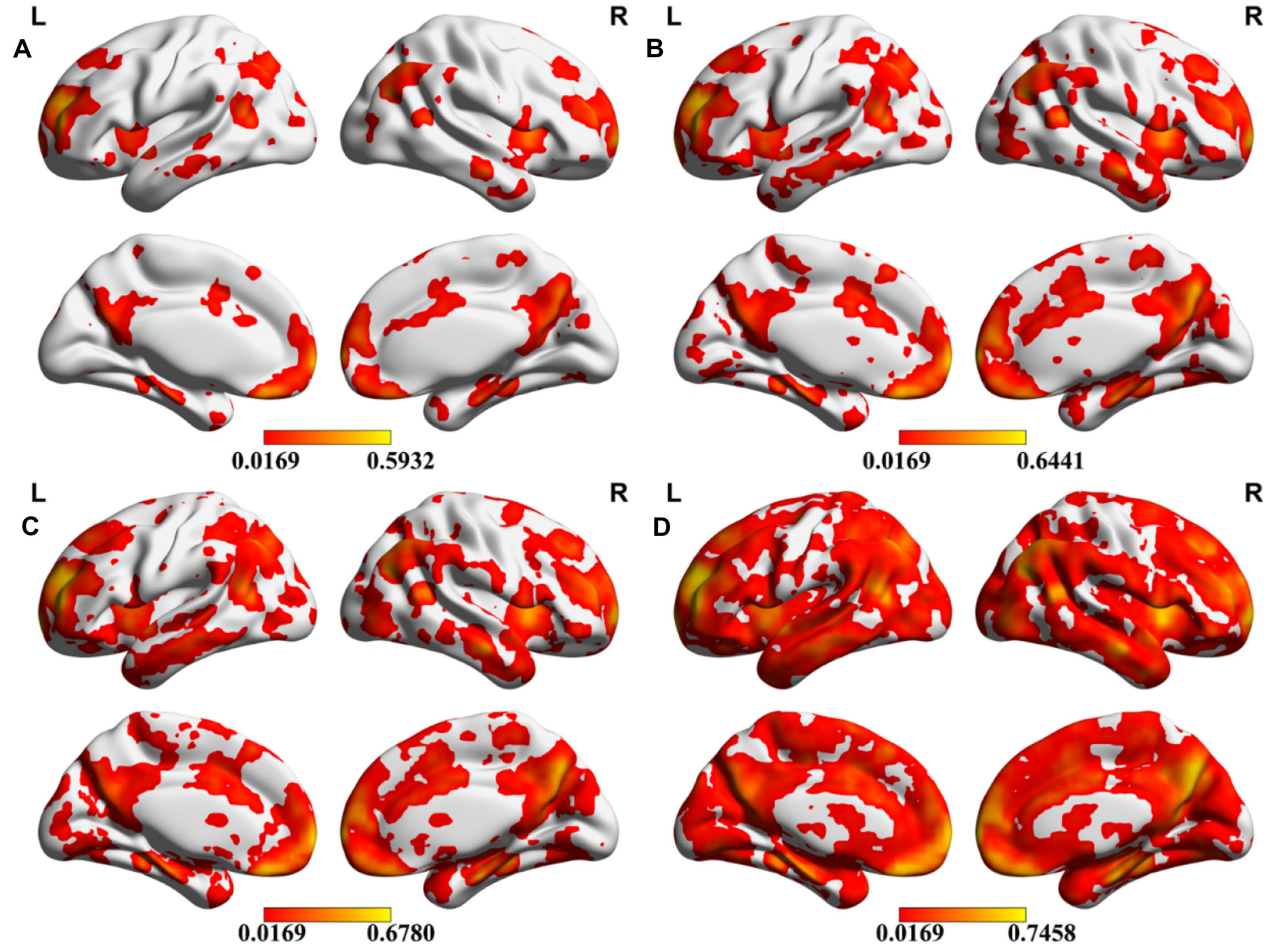
Overlap of different seeds. Meta-analytic results were transformed to binary images, and the percentage of each voxel was calculated after all binary images added together. The color bar indicates the overlap rate of each voxel. **A–D** present the results under four uncorrected thresholds of the meta-analysis (*p* = 0.0001, *p* = 0.0005, *p* = 0.001, *p* = 0.005).

**Figure 3:**
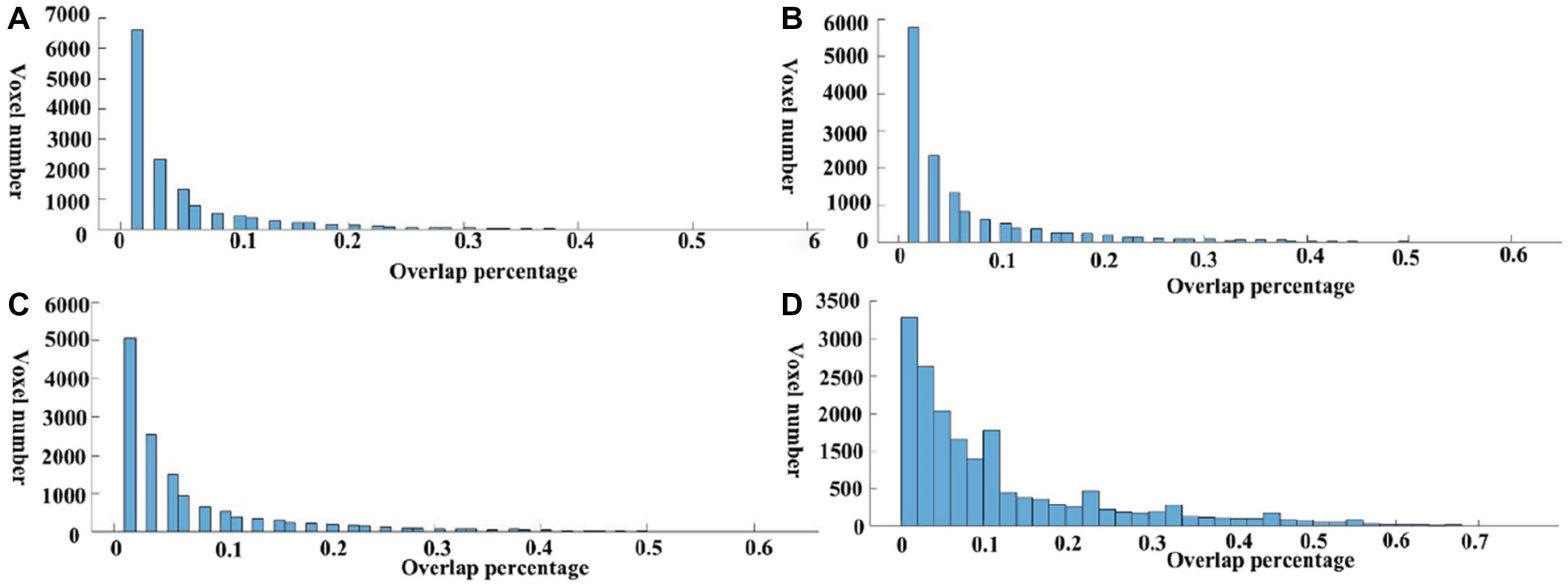
Overlap percentage of different seeds. The value of each voxel in the overlap maps was extracted, and the voxel number under each overlap percentage was calculated. **A–D** present the results under four uncorrected thresholds of the meta-analysis (*p* = 0.0001, *p* = 0.0005, *p* = 0.001, *p* = 0.005).

Meta-analysis can produce robust results and therefore be used here to investigate the reproducibility across different seeds. To evaluate the consistency of meta results among all DMN seeds, we calculated the overlap percentage of each voxel of all 59 meta-analytic brain images at four meta uncorrected *P* thresholds (Figure 1). Specifically, a binarized mask was created for each meta-analytic image. Further, we summed all the binary maps voxel-by-voxel and calculated an overlap rate map. Specifically, all the 59 meta-analytic maps firstly transformed into a binarized map which means that all the ono-zero values were transformed into 1 and zero values keep unchanged. For each voxel in the whole brain, the consistency value was calculated by dividing the sum of the values in all the 59 binarized maps by 59. The percentage of most voxels is less than 10% which showed very low overlap among these DMN seeds (Figures 2, 3). Compared with the whole brain voxel number, a minimal number of voxels exceed 50% regardless of the different thresholds of meta-analysis, which provide us with an intuitive distribution of the overlap among meta-analytic results.

### 4.3. Overlap between different brain regions of DMN

The Dice coefficient (Dice, 1945) was used to evaluate replicability between every two seeds of DMN, which is calculated by the formula:


$$Dice=\frac{2\times V_{overlap}}{V_{1}+V_{2}}$$

**Figure 4:**
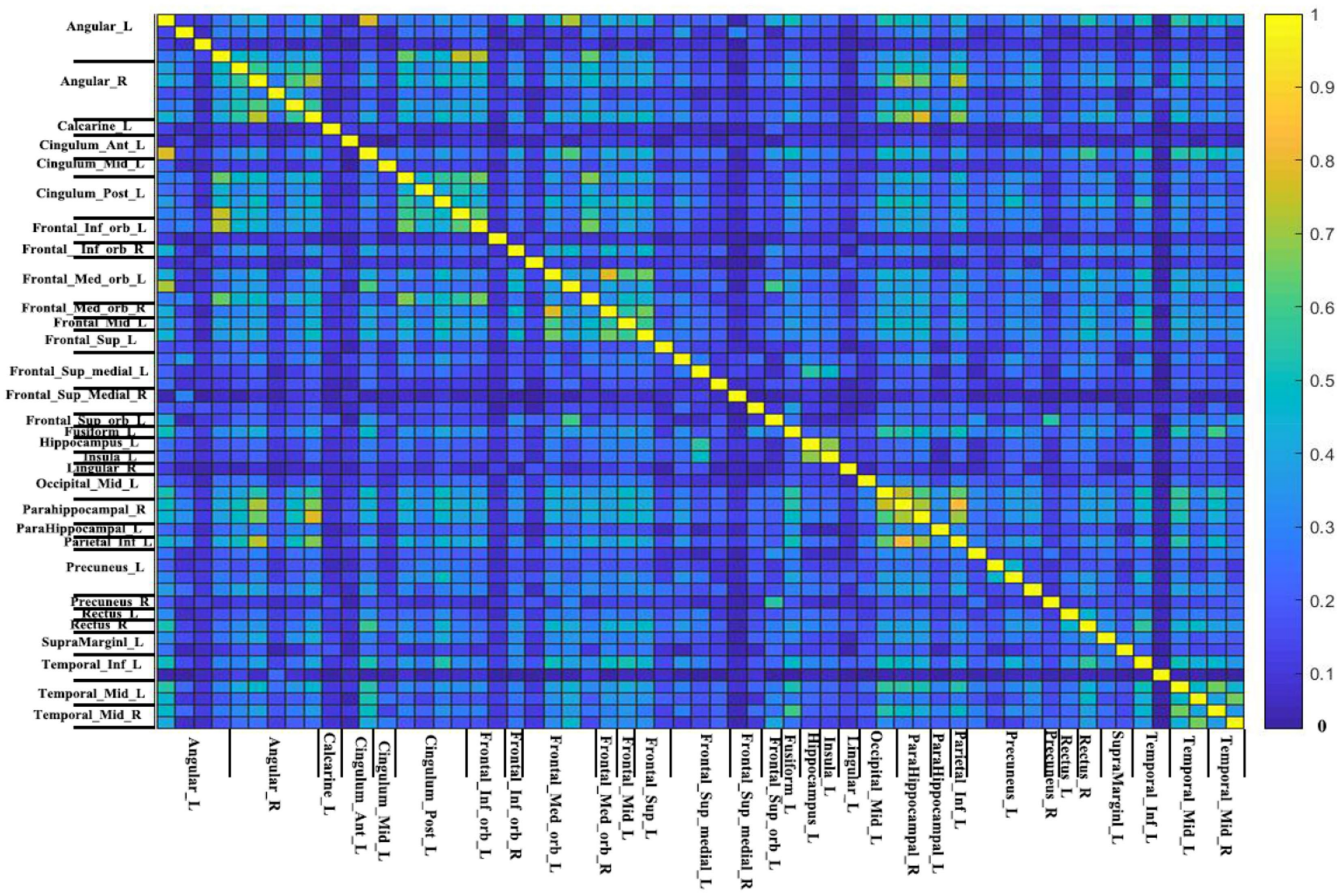
The Dice coefficient matrix. The threshold-free Dice coefficient matrix of each seed with all other seeds represent the overlap of every two meta-analytic maps. This is because the name of seeds between two short lines is the same (just different in location). The color bar indicates the Dice coefficient. The higher the Dice coefficient, the better the overlap.

V_1_ and V_2_ are the number of non-zero voxels in two IBMA images that are thresholded in the meta-analysis. V_overlap_ is the number of non-zero voxels in both images. Dice coefficient ranges from 0 to 1, in which 0 represents no overlap and 1 represents good overlap. Figure 4 displays the Dice coefficient matrix across different DMN seeds. It is observed that the overlap among these seeds is not very good in general.

**Figure 5:**
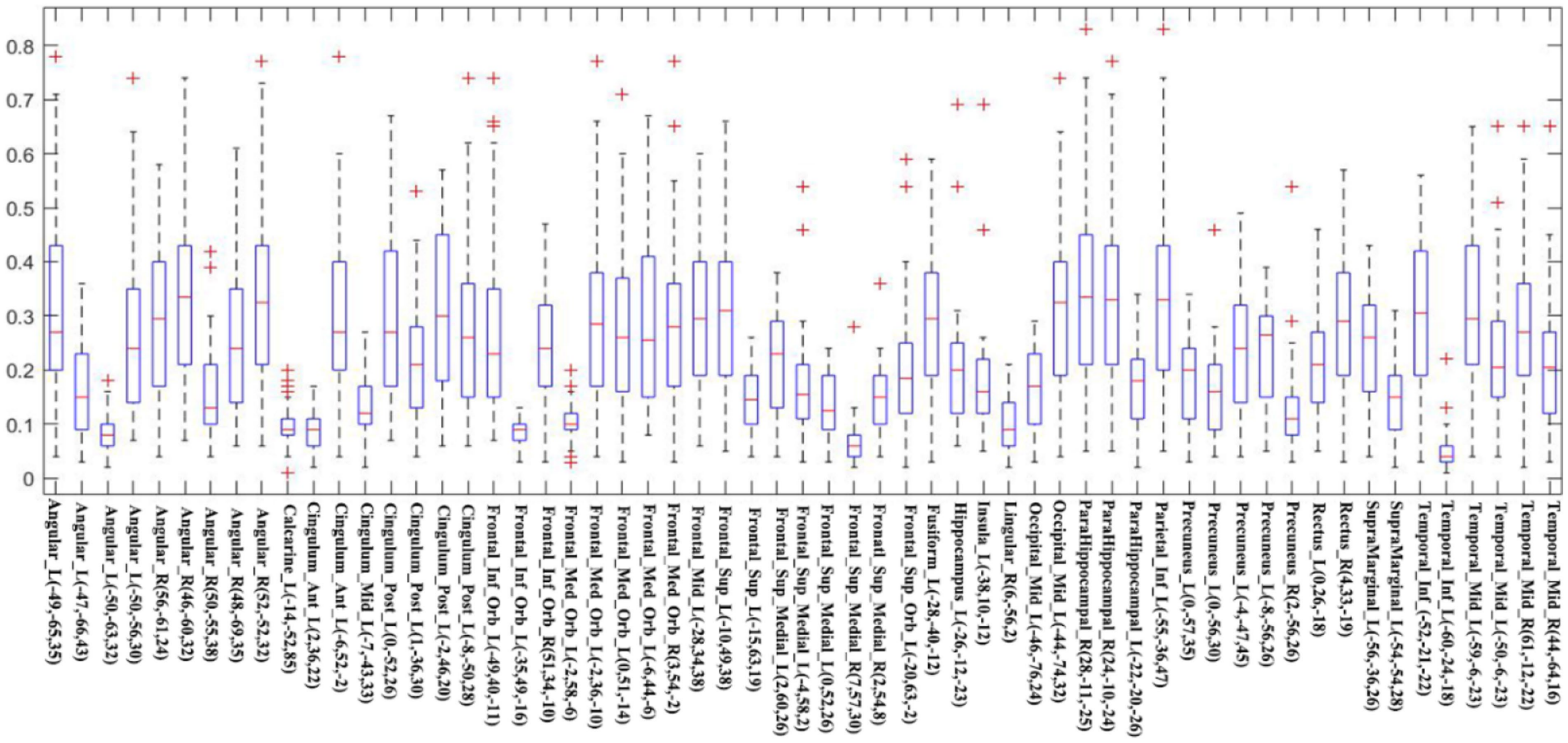
The Dice coefficient of each seed is shown using a boxplot. The red line in every box represents the median Dice coefficient, and the edges of the box represent the 25th and 75th percentiles. The whiskers extend to 1.5 interquartile range (IQR), and red “+” signs display values beyond 1.5 IQR. This note applies to the following Boxplots.

To measure the general overlap of different DMN brain regions without being affected by the extreme values, we calculated the median Dice coefficient of each seed (Figure 5). The median Dice coefficient of 59 DMN seeds was from 0.043 which belongs to Temporal_Inf_L (−60, −24, −18) to 0.337 originating from Angular_R (46, −60, 32). It also revealed a poor overlap among these seeds, illustrating in Figure 4 that the overlaps of different DMN seeds are quite different.

### 4.4. Classification of different DMN seeds

**Figure 6:**
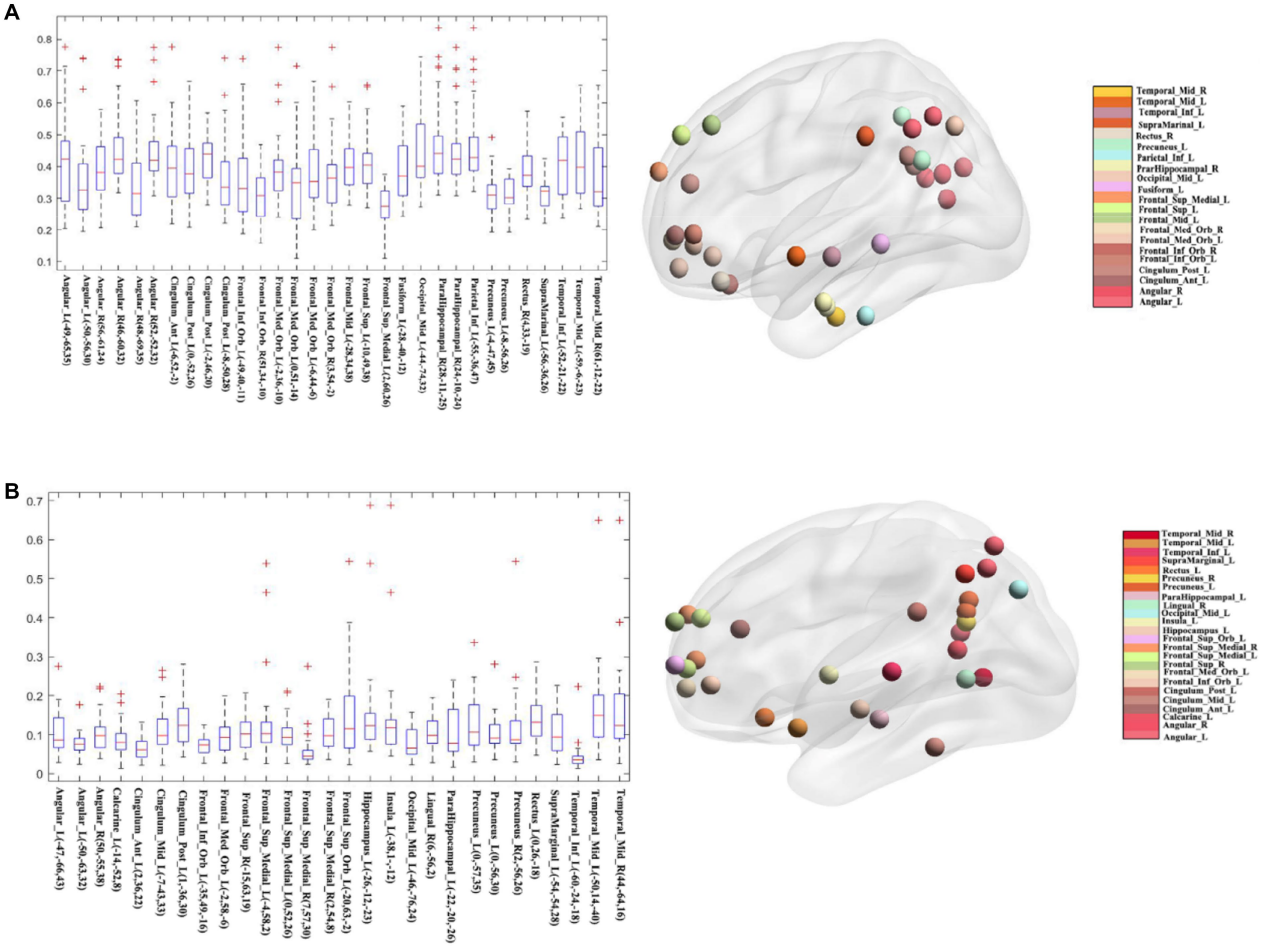
Classification of different seeds. **(A)** The seeds whose median Dice coefficient above average and their visualized network pattern. **(B)** The seeds whose median Dice coefficient below average and their visualized network pattern.

To figure out which seed yielded better overlap results, we further calculated the average of all medians and considered the seeds above average with a higher overlap than those below average (see Figure 6). As Figure 6 shows, the nodes have a higher median Dice coefficient with a relatively similar distribution. Therefore, we believe that the meta results of these seeds are much more consistent. And Figure 6B showed the distribution of seeds with a lower median Dice coefficient, which represents that these seeds showed lower overlap with other seeds belonging to DMN. The mean and median Dice coefficient of each seed was showed in the Table 3.

## 5. Discussion

Seed-based functional connectivity is one of the most widely used methods in resting-state fMRI studies. It measures the linear temporal correlation between the seed regions and every other voxel in the whole brain. Assessing seed selection arbitrariness on studies’ results, the current meta-analysis revealed that the results of seed-based functional connectivity were exclusively affected by choice of seed. This was accomplished using image-based meta-analysis to identify seeds with relatively high reproducibility due to their high sensitivity. We then performed several image-based meta-analyses with 59 different DMN seeds from the retrieved articles. Among these meta-analytic maps, a very low degree of spatial overlap has been displayed. The overlap rate of most voxels in the whole brain was less than 10%, and the median Dice coefficient of all 59 seeds fluctuated wildly.

Functional connectivity should be reliable, sensitive and specific to longitudinal changes (Dosenbach et al., 2010; Satterthwaite et al., 2014). However, the reliability of functional imaging results has been criticized in recent years (Bennett and Miller, 2010). Many studies have estimated the test–retest reliability of resting-state functional connectivity, and the results ranged from poor to good (Shehzad et al., 2009; Noble et al., 2017; Pannunzi et al., 2017). Our study supported the assumption that the reliability of functional connectivity across different seed ROIs is poor. Consistent with the finds of Noble et al. (2017), the different locations of seed region in previous studies lower the test–retest reliability of functional connectivity. Again, Wu et al. (2018) concluded that the seed-based functional connectivity patterns are unreliable. Although previous studies have proposed that different seed voxels produce different maps and the selection of seed regions would bias the results and restrict the functional connectivity map to the selected regions (Cordes et al., 2000; Van den Heuvel and Hulshoff Pol, 2010), our study provides quantitative evidence to support the proposition. There is a very low degree of overlap among all the meta-analytic results, indicating that different seeds cannot produce the same functional map.

Although our study selected seeds from classic articles, the low Dice coefficient speaks for relatively low reproducibility across meta-analytic results of different seeds. The arbitrary selection of seeds has been proven to influence functional connectivity results (Marrelec and Fransson, 2011). Even when the same strategy is used, the difference in location results in significant changes to the connectivity pattern in the DMN (Cole et al., 2010; Hayasaka and Laurienti, 2010). In addition to static functional connectivity, the study has found that the reproducibility in dynamic functional connectivity is not high (Zhang et al., 2018). All of these give us a wake-up call that the selection of seeds should be thought through Cole et al. (2010) found that even the slightest spatial difference of seeds can affect the spatial characteristics of the resting-state networks. Thus, the selection of seed seems to be quite substantial. Sohn et al. (2015) discovered that inappropriate seeds would bring lower calculated connectivity and higher variance. The overall replicability challenge is severe; therefore, any attempts to ascertain which seeds possess relatively higher reproducibility are a worthy pursuit. The dice coefficient matrix shows a very complex pattern of the overlap among these seeds in which some seeds generate a high Dice coefficient while some seeds are with low overlap. Different seeds yielded quite different results of reproducibility. Margulies et al. (2009) revealed that even a slight shift of seed location within the precuneus produced significant changes in the connectivity pattern. It is worth noting that the seeds with relatively high reproducibility should be treated with caution. They may show different results in different diseases.

Some limitations deserve further investigation in future work. First, considering the prominence of DMN in current literature, we targeted DMN rather than other resting-state networks. Thus, the reproducibility across different seeds in other resting-state networks needs further investigation. Second, our study chose the sex difference as the model to explore the statistical difference due to its stability compared with disease. Therefore, caution should be applied in interpreting our conclusions in the context of diseases. In respect of this, further research adopting a difference-in-disease model is needed. Third, due to lack of the psychiatric or neurologic history information of the participants, the exclusion criteria did not include the psychiatric or neurologic history. Thus, the interpretation of the results should be cautious. Fourth, although the present study revealed the poor reproducibility among different seed of the same network, future study should focus on finding out the robust seed.

## 6. Conclusion

This study demonstrates that the selection of seeds influences the functional connectivity pattern and generates inconsistent results among the seeds. Although previous studies have noted this issue, our study provides quantitative evidence through image-based meta-analysis. There was a very low degree of spatial overlap among these meta-analytic results. From this perspective, researchers need to be cautious in selecting the seed regions. Thus, special attention should be paid to the seeds with extremely low reproducibility illustrated in our study.

## Data availability statement

Publicly available datasets were analyzed in this study. This data can be found here: The datasets analyzed during the current study are available in the International Neuroimaging Data-sharing Initiative (INDI), Consortium for Reliability and Reproducibility (CoRR) — Consortium for Reliability and Reproducibility (CoRR) documentation.

## Author contributions

JR and X-ZJ designed the study. J-WS collected and analyzed the data. M-TL wrote the first draft of the manuscript, which JR, X-ZJ, Y-TL, CA, and L-LZ revised. All authors contributed to the article and approved the submitted version.

## Funding

This Funding was obtained through the Open Research Fund of College of Teacher Education, Zhejiang Normal University: Jykf22001w, National Natural Science Foundation of China: 82001898 and Beijing Well-being Foundation 2021 Key Project of Positive Psychology.

## Conflict of interest

The authors declare that the research was conducted in the absence of any commercial or financial relationships that could be construed as a potential conflict of interest.

## Publisher’s note

All claims expressed in this article are solely those of the authors and do not necessarily represent those of their affiliated organizations, or those of the publisher, the editors and the reviewers. Any product that may be evaluated in this article, or claim that may be made by its manufacturer, is not guaranteed or endorsed by the publisher.

## Footnotes

^1^ http://restfmri.net/CORR_meta_maps.zip
