# Supplementary material for: The effect of seed location on functional connectivity: evidence from an image-based meta-analysis
Source: Front Neurosci. 2023 May 31;17:1120741. doi: 10.3389/fnins.2023.1120741 (PMC10264592; doi:10.3389/fnins.2023.1120741)
Supplement: Supplementary file 1 [file Data_Sheet_1.docx]

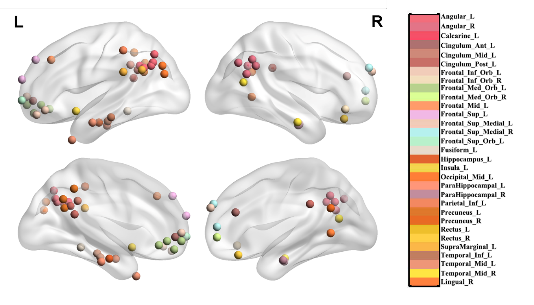


**Figure S1.** The Multiview of the location of al the seeds within the default mode network. Seeds with the same name are marked with the same color.


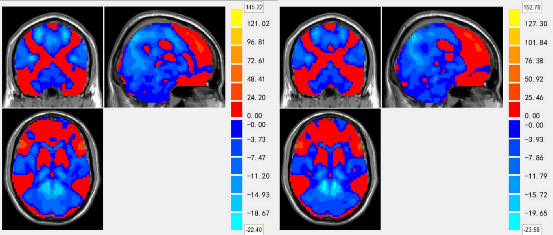


**Figure S2.** The one sample t -test maps of FC maps based on frontal_Inf_orb_R (46, 31, -1) (left for male, right for female)


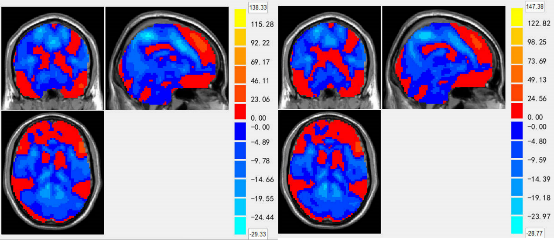


**Figure S3.** The one sample t -test maps of FC maps based on frontal_Inf_orb_L (-46, 37, -3) (left for male, right for female)


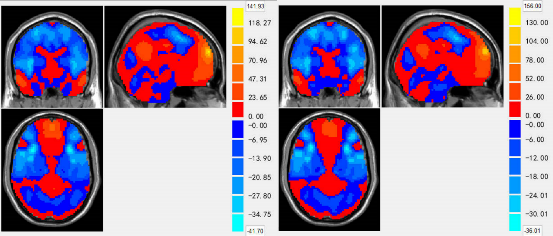


**Figure S4.** The one sample t -test maps of FC maps based on frontal_Sup_Medical_L (1, 52, 33) (left for male, right for female)


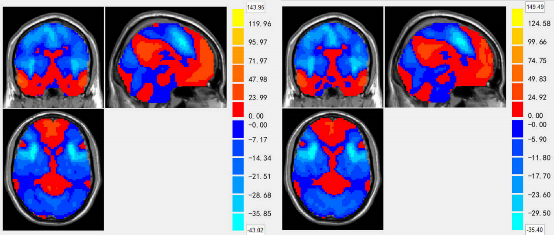


**Figure S5.** The one sample t -test maps of FC maps based on temporal_Mid_R (56, -11, -16) (left for male, right for female)


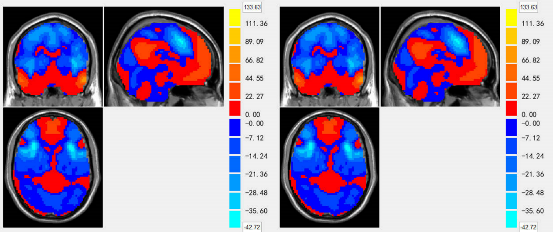


**Figure S6.** The one sample t -test maps of FC maps based on temporal_Mid_L (-55, -5, -18) (left for male, right for female)


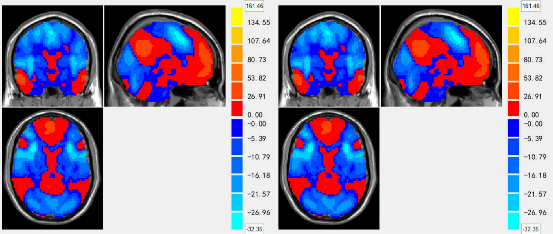


**Figure S7.** The one sample t -test maps of FC maps based on Angular_R (50, -61, 21) (left for male, right for female)


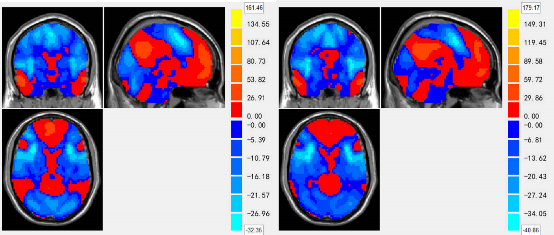


**Figure S8.** The one sample t -test maps of FC maps based on Angular_L (-47, -65, 29) (left for male, right for female)


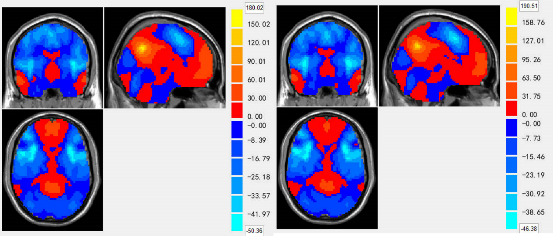


**Figure S9.** The one sample t -test maps of FC maps based on Precuneus_L (-2, -58, 30) (left for male, right for female)


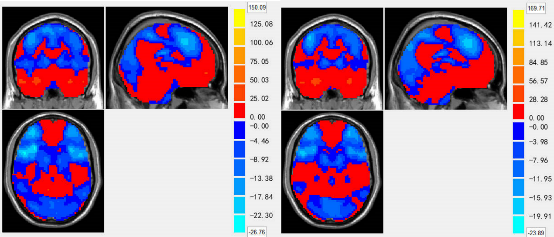


**Figure S10.** The one sample t -test maps of FC maps based on paraHippocampal (25, -10, -19) (left for male, right for female)


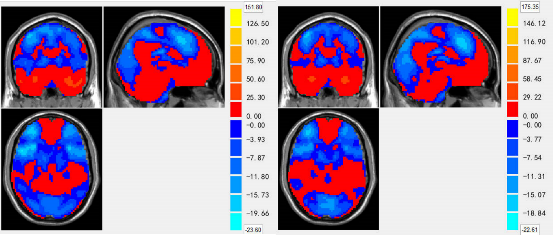


**Figure S11.** The one sample t -test maps of FC maps based on hippocampus_L (-25, -11, -18) (left for male, right for female)


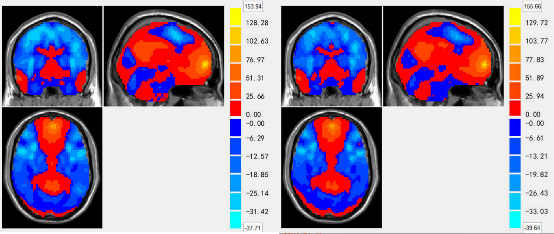


**Figure S12.** The one sample t -test maps of FC maps based on frontal_Sup_Medial_L (-4, 58, 2) (left for male, right for female)


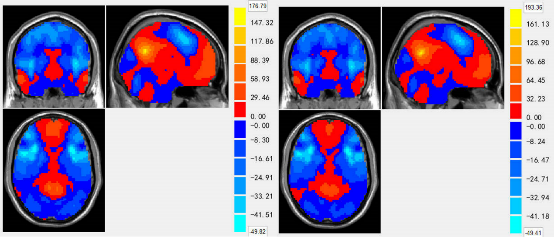


**Figure S13.** The one sample t -test maps of FC maps based on precuneus_R (-2, -56, 26) (left for male, right for female)


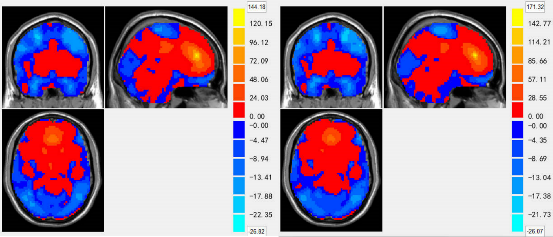


**Figure S14.** The one sample t -test maps of FC maps based on cingulum_Ant_L (2, 36, 22) (left for male, right for female)


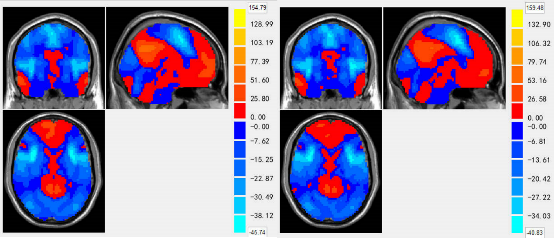


**Figure S15.** The one sample t -test maps of FC maps based on angular_R (46, -60, 32) (left for male, right for female)


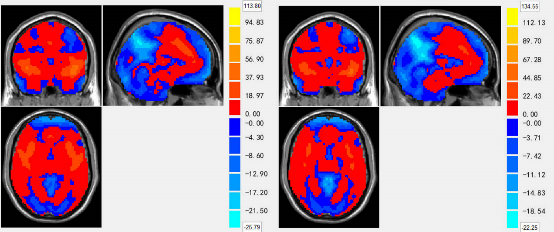


**Figure S16.** The one sample t -test maps of FC maps based on insula_L (-38, 10, -12) (left for male, right for female)


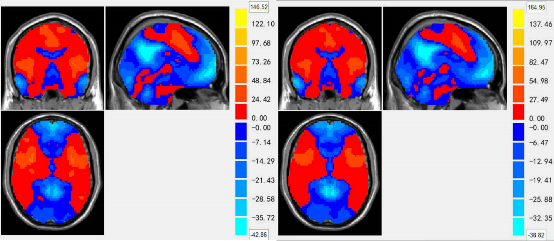


**Figure S17.** The one sample t -test maps of FC maps based on supraMaraginal_L (-56, -36, 26) (left for male, right for female)


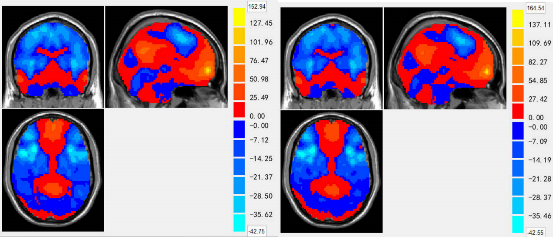


**Figure S18.** The one sample t -test maps of FC maps based on frontal_Med_Orb_L (-2, 56, -6) (left for male, right for female)


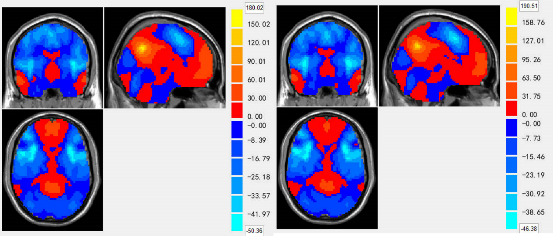


**Figure S19.** The one sample t -test maps of FC maps based on lingual_R (-6, -56, 2) (left for male, right for female)


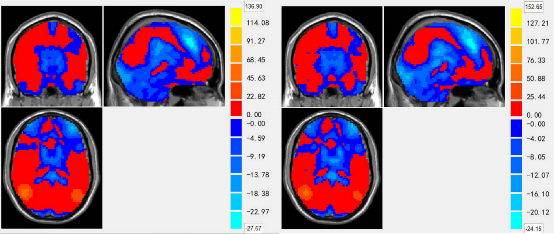


**Figure S20.** The one sample t -test maps of FC maps based on temporal_Mid_R (44, -64, 16) (left for male, right for female)


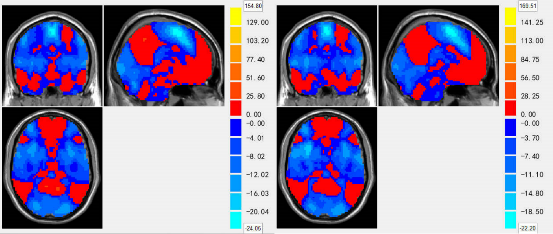


**Figure S21.** The one sample t -test maps of FC maps based on occipital_Mid_L (-46, -76, 24) (left for male, right for female)


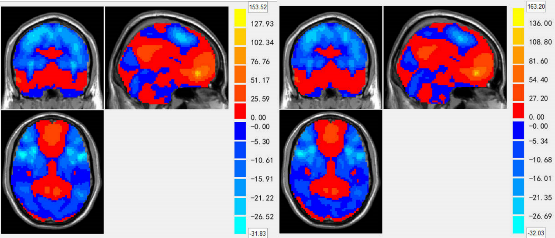


**Figure S22.** The one sample t -test maps of FC maps based on frontal_Med_Orb_L (-2, 36, -10) (left for male, right for female)


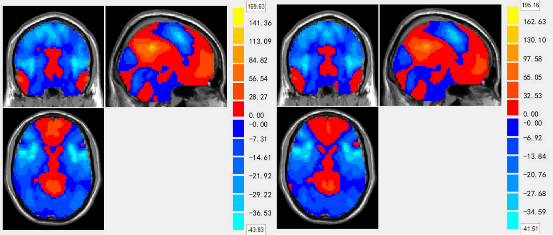


**Figure S23.** The one sample t -test maps of FC maps based on cingulum_Mid_L (--7, -43, 33) (left for male, right for female)


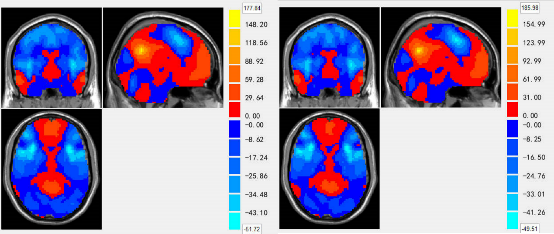


**Figure S24.** The one sample t -test maps of FC maps based on precuneus_L (0, -56, 30) (left for male, right for female)


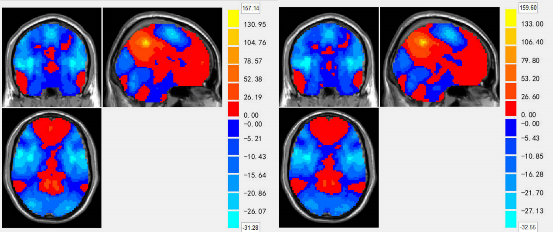


**Figure S25.** The one sample t -test maps of FC maps based on precuneus_L (-4, -47, 45) (left for male, right for female)


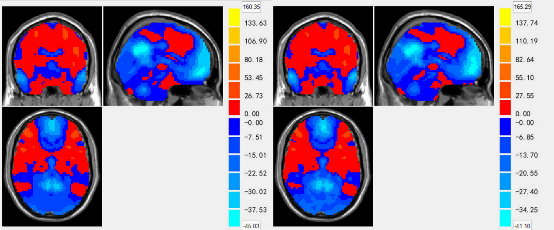


**Figure S26.** The one sample t -test maps of FC maps based on parietal_Inf_L (-55, -36, 47) (left for male, right for female)


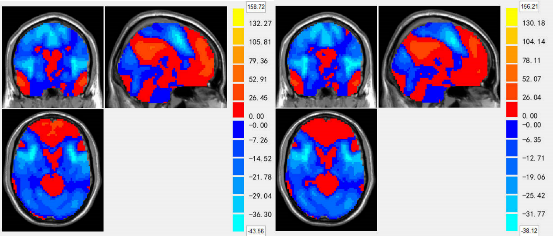


**Figure S27.** The one sample t -test maps of FC maps based on angular_L (-46, -66, 43) (left for male, right for female)


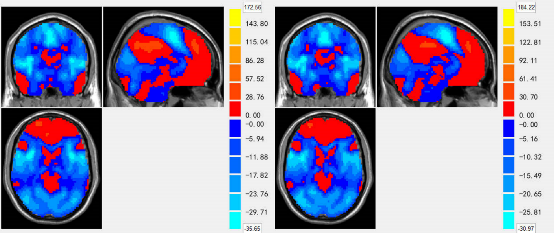


**Figure S28.** The one sample t -test maps of FC maps based on angular_R (50, -55, 38) (left for male, right for female)


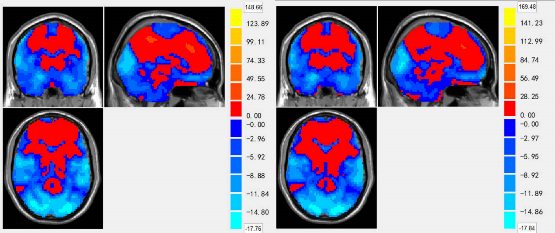


**Figure S29.** The one sample t -test maps of FC maps based on frontal_Mid_L (-27, 34, 37) (left for male, right for female)


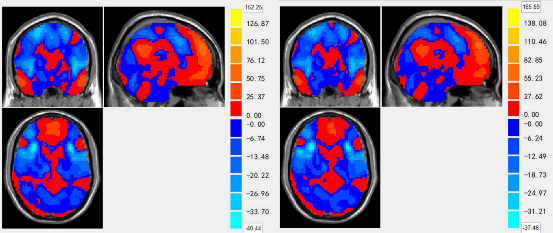


**Figure S30.** The one sample t -test maps of FC maps based on frontal_Sup_L (-10, 49, 37) (left for male, right for female)


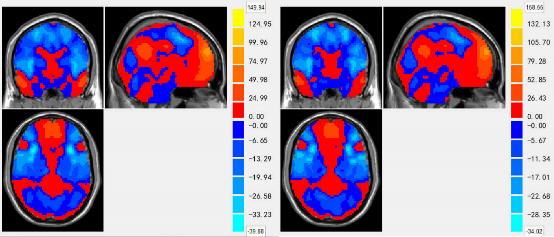


**Figure S31.** The one sample t -test maps of FC maps based on frontal_Sup_Medial_R (6, 57, 29) (left for male, right for female)


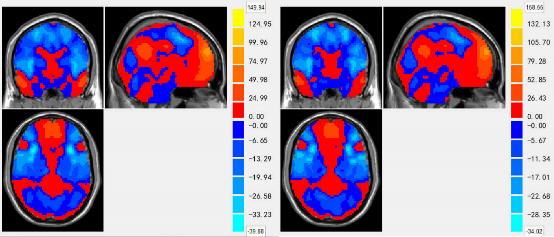


**Figure S32.** The one sample t -test maps of FC maps based on frontal_Sup_L (-15, 62, 18) (left for male, right for female)


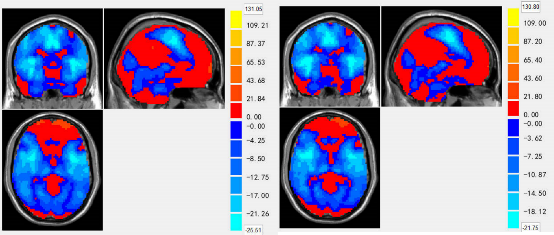


**Figure S33.** The one sample t -test maps of FC maps based on frontal_Sup_Orb_L (-19, 62, -1) (left for male, right for female)


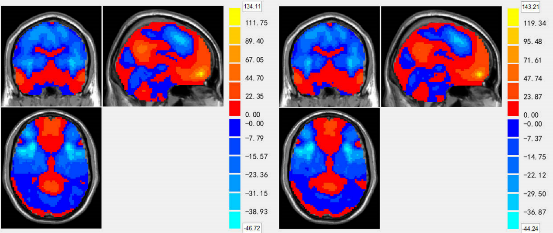


**Figure S34.** The one sample t -test maps of FC maps based on frontal_Med_Orb_L (0, 51, -14) (left for male, right for female)


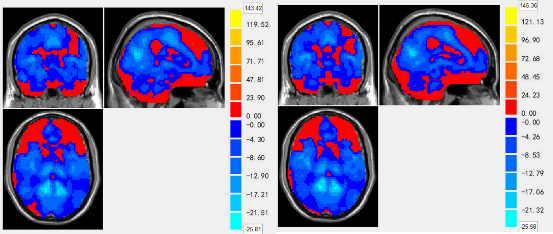


**Figure S35.** The one sample t -test maps of FC maps based on frontal_Inf_Orb_L (-34, 48, -15) (left for male, right for female)


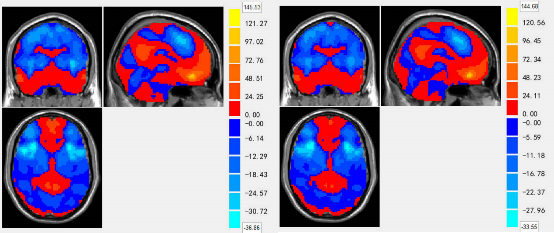


**Figure S36.** The one sample t -test maps of FC maps based on rectus_R (4, 33, -19) (left for male, right for female)


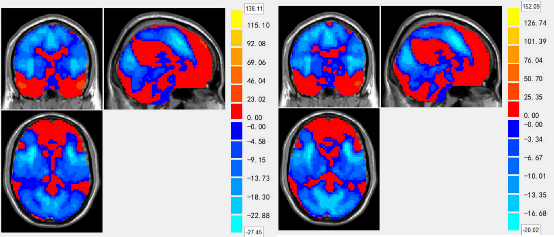


**Figure S37.** The one sample t -test maps of FC maps based on temporal_Inf_L (-52, -20, -21) (left for male, right for female)


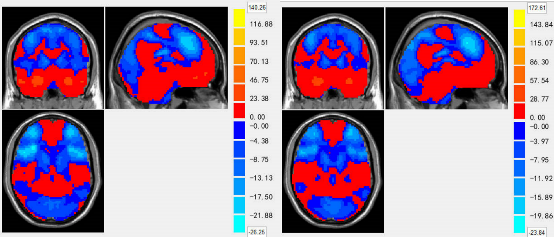


**Figure S38.** The one sample t -test maps of FC maps based on paraHippocampal_R (23, -9, -24) (left for male, right for female)


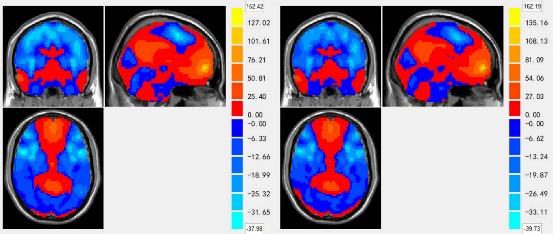


**Figure S39.** The one sample t -test maps of FC maps based on frontal_Med_Orb_R (3, 54, -2) (left for male, right for female)


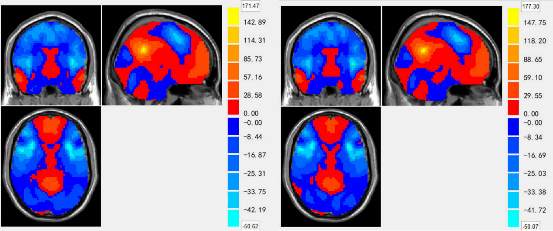


**Figure S40.** The one sample t -test maps of FC maps based on cingulum_Post_L (0, -52, 26) (left for male, right for female)


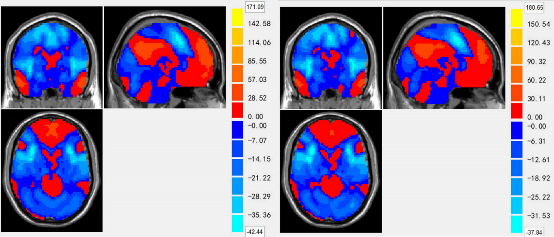


**Figure S41.** The one sample t -test maps of FC maps based on angular_L (-50, -63, 32) (left for male, right for female)


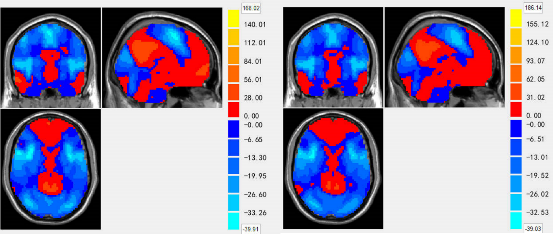


**Figure S42.** The one sample t -test maps of FC maps based on angular_R (48,-69, 35) (left for male, right for female)


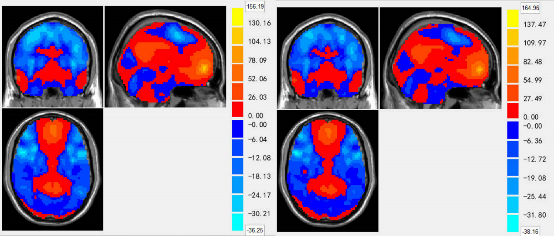


**Figure S43.** The one sample t -test maps of FC maps based on cingulum_Ant_L (-6, 52, -2) (left for male, right for female)


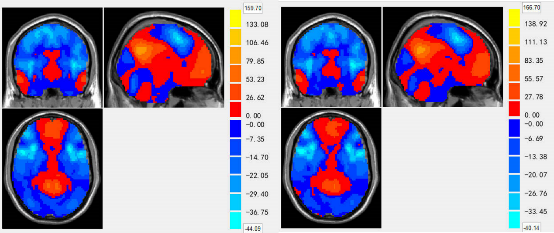


**Figure S44.** The one sample t -test maps of FC maps based on precuneus_L (-8, -56, 26) (left for male, right for female)


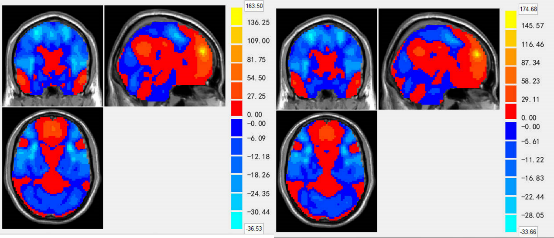


**Figure S45.** The one sample t -test maps of FC maps based on frontal_Sup_Medial_L (0, 52, 26) (left for male, right for female)


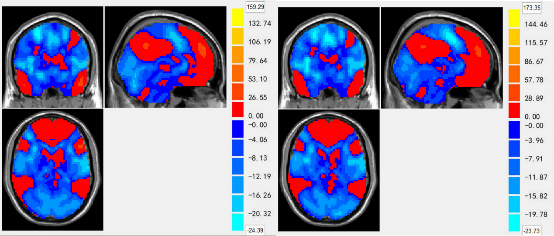


**Figure S46.** The one sample t -test maps of FC maps based on supraMarginal_L (-54, -54, 28) (left for male, right for female)


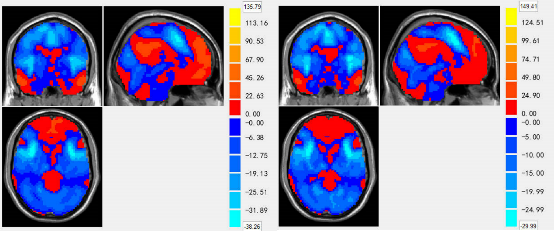


**Figure S47.** The one sample t -test maps of FC maps based on temporal_Inf_L (-60, -24, -18) (left for male, right for female)


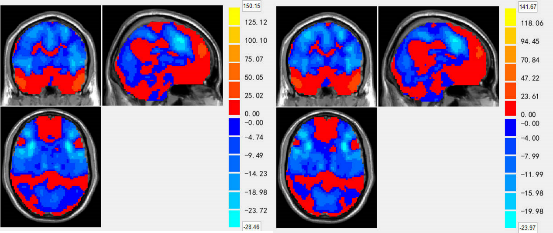


**Figure S48.** The one sample t -test maps of FC maps based on temporal_Med_L (-50, 14, -40) (left for male, right for female)


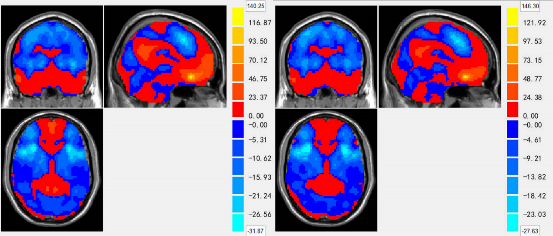


**Figure S49.** The one sample t -test maps of FC maps based on rectus_L (0, 26, -18) (left for male, right for female)


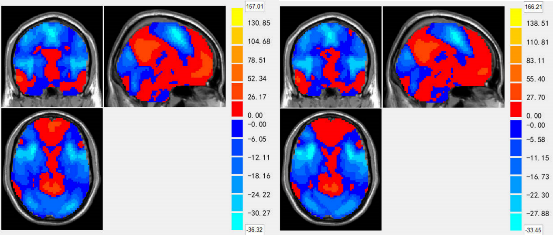


**Figure S50.** The one sample t -test maps of FC maps based on occipital_Mid_L (-44, -74, 32) (left for male, right for female)


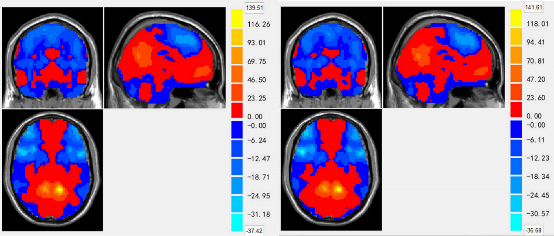


**Figure S51.** The one sample t -test maps of FC maps based on calcarine_L (-14, -52, 8) (left for male, right for female)


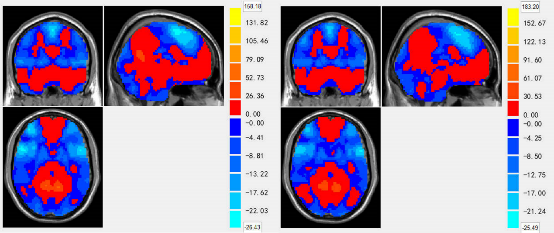


**Figure S52.** The one sample t -test maps of FC maps based on fusiform_L (-28, -40, -12) (left for male, right for female)


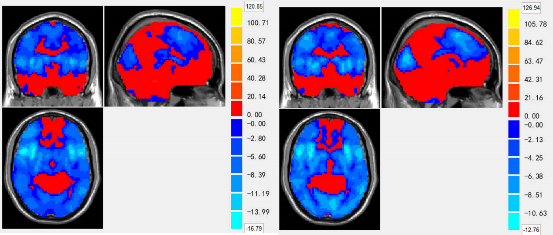


**Figure S53.** The one sample t -test maps of FC maps based on paraHippocampal (-22, -20, -26) (left for male, right for female)


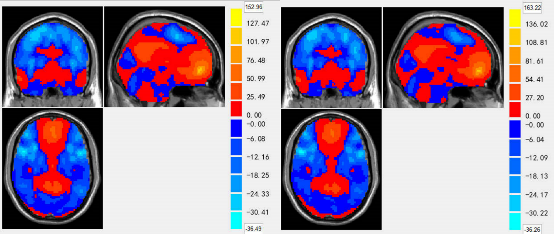


**Figure S54.** The one sample t -test maps of FC maps based on frontal_Med_Orb_L (6, 44, -6) (left for male, right for female)


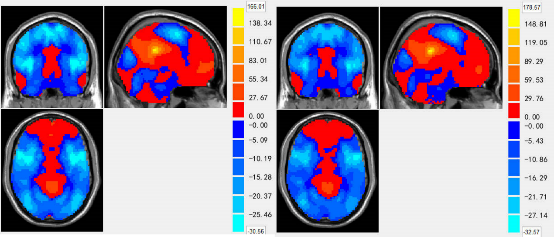


**Figure S55.** The one sample t -test maps of FC maps based on cingulum_Post_L (1, -36, 30) (left for male, right for female)


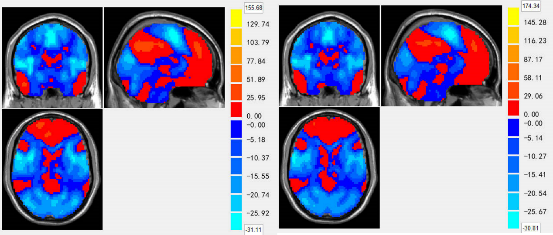


**Figure S56.** The one sample t -test maps of FC maps based on angular_R (52, -52, 32) (left for male, right for female)


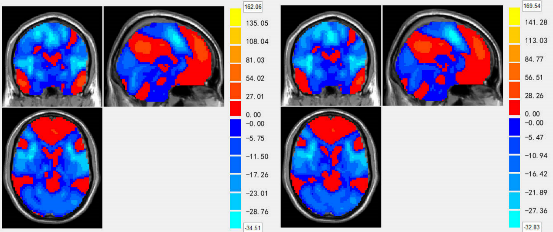


**Figure S57.** The one sample t -test maps of FC maps based on angular_L (-50, -56, 30) (left for male, right for female)


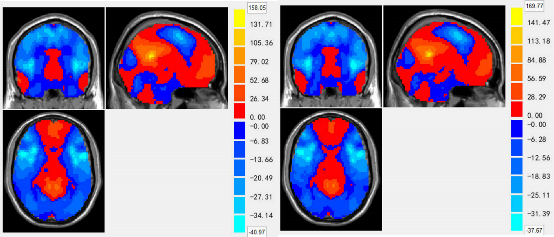


**Figure S58.** The one sample t -test maps of FC maps based on cingulum_Post_L (-2, -46, 20) (left for male, right for female)


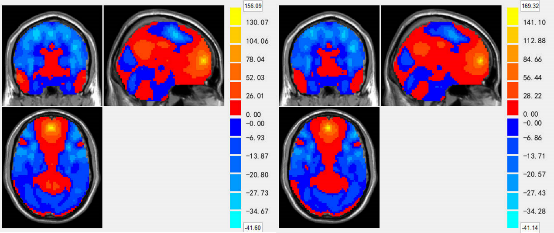


**Figure S59.** The one sample t -test maps of FC maps based on fronal_Sup_Medial_L (2, 54, 8) (left for male, right for female)


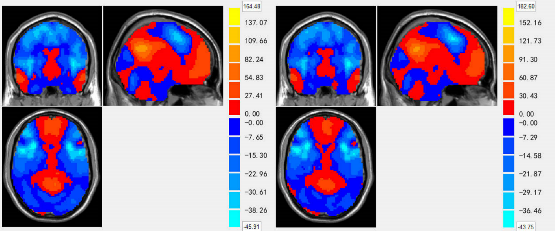


**Figure S60.** The one sample t -test maps of FC maps based on cingulum_Post_L (-8, -50, 28) (left for male, right for female)
